# Sustainability of Technical and Vocational Education and Training (TVET) along with Vocational Psychology

**DOI:** 10.3390/bs14100859

**Published:** 2024-09-24

**Authors:** Jian-Hong Ye, Zhen He, Bin Bai, Yu-Feng Wu

**Affiliations:** 1 Faculty of Education, Beijing Normal University, Beijing 100875, China; jianhong.ye@bnu.edu.cn (J.-H.Y.); hezhen@bnu.edu.cn (Z.H.); 2 National Institute of Vocational Education, Beijing Normal University, Beijing 100875, China; 3 Office of Physical Education, Ming Chi University of Technology, New Taipei City 24301, Taiwan

Since the first Industrial Revolution in the 18th century, the gap for technical talent began to widen, and the concept of large-scale technical and vocational education and training (TVET) began to be advocated. The formal TVET system also gradually took shape. TVET helps students develop or enhance their knowledge, skills, and abilities for specific occupations [1]. Unlike general education, vocational education is employment-oriented. Vocational education cultivates a group of students with high professional quality [2]. TVET includes technical education, vocational education, vocational training, on-the-job training, and apprenticeship training [3]. In every stage of the Industrial Revolution, TVET has been entrusted with the important role of talent cultivation. Therefore, the implementation and sustainable development of technical and vocational education and training have received attention from educational experts and governments worldwide. To embrace the new changes in technical and vocational education and training, research and exploration in this field are particularly important. At the same time, as a significant branch of study, psychology theories based on vocational education should also be given due attention. This is closely related to talent cultivation, formal vocational education, informal vocational education, career development, and career planning. In addition, over the past century, the establishment of the TVET system has been widely regarded as crucial for the industrial and economic development of countries (regions) and cities, promoting employment, addressing livelihood issues, and implementing educational equity.

Sustainable Development Goal 4 emphasizes creating lifelong learning opportunities through high-quality technical and vocational education and imparting the necessary practical skills [4]. Today, vocational education is no longer as simple as the technical education it was at its inception. With the call for lifelong education and competency-based education, in addition to cultivating high-level vocational skills, TVET also focuses on how to help learners develop into “well-rounded individuals.” Experts, scholars, and educators are dedicated to exploring how to cultivate 21st-century competencies in vocational school students. However, in TVET, critical thinking is difficult to cultivate because acquiring practical skills is often considered the primary focus in technical and vocational education and training [5]. However, professional competence can be divided into knowledge, skills, emotions, attitudes, and values. Therefore, in modern TVET, while teaching students, emphasis is placed not only on knowledge retention and comprehension, but also on knowledge application, knowledge transfer, and the integration of interdisciplinary knowledge. Additionally, how teachers design effective instruction for students to engage in effective learning, as well as discussions on interdisciplinary learning, multimodal learning, technology-assisted learning, and meaningful learning in vocational education, are also important topics. Another important feature of TVET is that students need to continuously engage in hands-on practice. Besides deliberate practice, another key educational characteristic is meaningful error-based learning, which allows TVET students to reflect on, internalize, and adjust from their error experiences.

In addition, continuous technological innovation and transformation have changed human society and the development of civilization, further promoting the high-quality development of productivity across various industries. As we gradually enter the Industry 4.0 era that began in the 2010s, the intelligent upgrading of industries, automation, robotics, artificial intelligence, and other technologies are bringing transformative changes to industries. This has led to the disappearance of some traditional jobs, with many labor-intensive occupations being replaced or having their original job content altered. Consequently, there have been groundbreaking changes in the demand for talent. The digital era has had a profound impact on industry positions. People’s anxiety about their jobs being replaced is becoming increasingly evident. It is also evident that intelligent manufacturing and digital transformation are strongly driving the development and change of vocational education and training. This not only accelerates the high-quality construction of higher vocational education, but also makes the cultivation of high-level skilled and innovative talents, as required for the 21st century, a new goal for TVET. This has also led to significant attention being paid to topics such as the development of emerging professions, digital literacy, talent cultivation mechanisms for digital occupations, 21st-century core competencies for vocational school students, and career development pathways. At the same time, exploring how digital technologies can promote the modernization of education, and how TVET can advance educational equity and lifelong learning practices, are crucial topics. This includes research on online vocational education, continuing education, the integration of different educational stages, educational transition mechanisms, lifelong (elderly) universities, credit banks, and national/regional qualification frameworks, which are also important global research hotspots of this century. In addition, continuous technological innovation and transformation have changed human society and the development of civilization, further promoting the high-quality development of productivity across various industries. As we gradually enter the Industry 4.0 era that began in the 2010s, the intelligent upgrading of industries, automation, robotics, artificial intelligence, and other technologies are bringing transformative changes to industries. This has led to the disappearance of some traditional jobs, with many labor-intensive occupations being replaced or having their original job content altered. Consequently, there have been groundbreaking changes in the demand for talent. The digital era has had a profound impact on industry positions. People’s anxiety about their jobs being replaced is becoming increasingly evident. It is also evident that intelligent manufacturing and digital transformation are strongly driving the development and change of vocational education and training. This not only accelerates the high-quality construction of higher vocational education, but also makes the cultivation of high-level skilled and innovative talents, as required for the 21st century, a new goal for TVET. This has also led to significant attention being paid to topics such as the development of emerging professions, digital literacy, talent cultivation mechanisms for digital occupations, 21st-century core competencies for vocational school students, and career development pathways. At the same time, exploring how digital technologies can promote the modernization of education, and how TVET can advance educational equity and lifelong learning practices, are crucial topics. This includes research on online vocational education, continuing education, the integration of different educational stages, educational transition mechanisms, lifelong (elderly) universities, credit banks, and national/regional qualification frameworks, which are also important global research hotspots of this century. 

Of course, the high-quality development of TVET cannot be achieved without the teacher workforce. Teachers, as frontline workers in the educational field, are the closest individuals to students within the school environment. As education evolves in response to new demands, the role of teachers is also changing [6]. Therefore, teacher training and the development of the teaching workforce are also crucial topics. This includes the preparation of pre-service teachers, the professional development of in-service teachers, and the progression from novice to experienced teachers and from novice to expert teachers. Issues such as teachers’ career development, leadership skills, professional adaptability, well-being, 21st-century competencies, educational spirit, industry internships for teachers, industry–academia cooperation, enhancement of teaching and academic skills, academic communities for teachers, and the development of teaching capabilities for enterprise-based teachers also require our attention.

Additionally, within the theme of “Sustainability of Technical and Vocational Education and Training (TVET) along with Vocational Psychology”, there are many research topics worth referencing, including the development of models to assess higher-order thinking skills in vocational education mathematics curricula, using system dynamics analysis to predict the achievement rates of school facilities and infrastructure, assessing the sustainability of TVET for construction management positions, the role of cultural capital in teacher–student interactions in Chinese vocational schools, challenges and strategies for the high-quality development of TVET in Germany, the acceptance of online learning by vocational school students in the context of COVID-19, factors influencing vocational teachers’ acceptance and use of information and communication technology in teaching, research on factors affecting TVET teachers’ professional competence, and the direct impact of fine motor training on the coordination and flexibility of the non-dominant hand in healthy adults. These research findings have expanded our understanding of the TVET field.

Although TVET is considered an important and irreplaceable educational system, research on TVET internationally still constitutes a relatively small proportion of research within the entire field of education. Considering the important role of TVET in the education, society, economy, and industrial development of various countries or regions, analyzing how to effectively cultivate practical and applicable skilled talents (technical experts) that meet the needs of nations (regions), enterprises, and society has always been a focal point of scholarly attention. Therefore, research on vocational education in specific countries or regions is also very important. For example, China has built the largest TVET system in the world, with unique characteristics and experiences that are worth learning from. This includes how vocational education in China can promote “new quality productivity (新质生产力)”. Topics such as the implementation and effectiveness of vocational education based on ideological and political education, the development of the teaching and academic skills of vocational school teachers, the new Level 8 worker system, the “Three Educations” (teachers, teaching materials, teaching methods) reform, national strength construction, rural vocational education, special vocational education, the role of vocational education in rural revitalization, the development of technological universities, graduate training within the TVET system, the integration of industry and education, work–study integration (work learning), the Luban Workshop (鲁班工坊), regional/area-specific educational practices, international cooperation and development in vocational education, the cultivation of master craftsmen (大国工匠) and their spirit, labor (awareness) spirit, the enhancement of the status of vocational education, field engineers, the inheritance and innovative transformation of intangible cultural heritage skills, vocational school skill competitions, the construction of vocational education academic communities, the construction of industry–education integration communities, the construction of industry–education integration alliances (产教融合), development pathways that establish mutual connections between vocational education and general education (职普融通), building a skills-based society, and building a learning-oriented society are all considered important topics for the high-quality development of contemporary vocational education in China, and are also of global interest in the education sector.

Additionally, how vocational education can effectively implement the United Nations’ Sustainable Development Goals will require further exploration of TVET practices and future development directions across different regions, countries, or areas. This includes education policies, vocational teacher training, career orientation education, educational systems, education regulations, talent cultivation mechanisms, school-enterprise cooperation, technological empowerment, technological inheritance and innovation, social evaluation of vocational education, curriculum and design, educational ecosystems, and knowledge frameworks. The aim is to develop localized TVET theories to ensure sustainable and high-quality development of vocational education. Of course, educational philosophy underpins educational theory, guiding our perspectives on the ideas and goals of TVET education. Therefore, exploring the philosophy of TVET can help stakeholders in vocational education deepen their understanding of the essence, purpose, value, process, and evaluation of TVET, thereby promoting the development of higher-quality vocational education. Therefore, research on the philosophy of TVET should be continuously conducted in conjunction with changes in the contemporary context.

Although vocational education and training (VET) reforms are becoming more common, there is more evidence regarding designing these reforms than implementing them [7]. Therefore, we call for future research on TVET to be strengthened and deepened from the perspectives of education, economics, management, psychology, sociology, behavioral science, policy studies, and other disciplines/multidisciplinary/interdisciplinary fields. This includes research topics such as the PISA Vocational Education and Training (VET) Assessment and analytical framework, cross-disciplinary integration skills, lifelong learning capabilities, problem discovery and solving abilities, forward thinking, innovative thinking, critical thinking, collaborative awareness, psychological skills, vocational leadership, and moral sense. It also involves student-centered learning methods such as lifelong learning, self-directed learning, personalized learning, cooperative learning, project-based learning, problem-based learning, inquiry-based learning, multimodal learning, and interdisciplinary learning based on curriculum ideological and political education (IP-STEAM). Additionally, there is a need to explore traditional apprenticeship systems, cognitive apprenticeships, modern apprenticeships, the development of new teaching materials, vocational competency assessment, social identity, social evaluation transformation, gender imbalance in vocational fields, competency gap analysis, vocational identity, career choice, career confidence, career preparation, job substitution, career development, and career adaptation, as well as industry skills, digital skills, interdisciplinary knowledge, internship quality, internship–learning balance, academic distress, academic counseling, competition coaching, bridging the gap between learning and application, the impact of involution on the development of education (teachers and students), international vocational education exchanges, and artificial intelligence in TVET. These research topics require ongoing exploration to provide a reference for experts, scholars, and stakeholders worldwide who are concerned about vocational education.

## Data Availability

This article does not include data.
